# Trends of *Mycobacterium bovis* Isolation and First-Line Anti-tuberculosis Drug Susceptibility Profile: A Fifteen-Year Laboratory-Based Surveillance

**DOI:** 10.1371/journal.pntd.0004124

**Published:** 2015-09-30

**Authors:** Miriam Bobadilla-del Valle, Pedro Torres-González, Miguel Enrique Cervera-Hernández, Areli Martínez-Gamboa, Brenda Crabtree-Ramirez, Bárbara Chávez-Mazari, Narciso Ortiz-Conchi, Luis Rodríguez-Cruz, Axel Cervantes-Sánchez, Tomasa Gudiño-Enríquez, Carmen Cinta-Severo, José Sifuentes-Osornio, Alfredo Ponce de León

**Affiliations:** 1 Department of Infectious Diseases, Laboratory of Clinical Microbiology, Instituto Nacional de Ciencias Médicas y Nutrición Salvador Zubirán, Mexico City, México; 2 Department of Infectious Diseases, HIV Clinic, Instituto Nacional de Ciencias Médicas y Nutrición Salvador Zubirán, Mexico City, Mexico; 3 Department of Medicine, Instituto Nacional de Ciencias Médicas y Nutrición Salvador Zubirán, Mexico City, Mexico; Swiss Tropical and Public Health Institute, SWITZERLAND

## Abstract

**Background:**

*Mycobacterium tuberculosis* causes the majority of tuberculosis (TB) cases in humans; however, in developing countries, human TB caused by *M*. *bovis* may be frequent but undetected. Human TB caused by M. *bovis* is considered a zoonosis; transmission is mainly through consumption of unpasteurized dairy products, and it is less frequently attributed to animal-to-human or human-to-human contact. We describe the trends of *M*. *bovis* isolation from human samples and first-line drug susceptibility during a 15-year period in a referral laboratory located in a tertiary care hospital in Mexico City.

**Methodology/Principal Findings:**

Data on mycobacterial isolates from human clinical samples were retrieved from the laboratory’s database for the 2000–2014 period. Susceptibility to first-line drugs: rifampin, isoniazid, streptomycin (STR) and ethambutol was determined. We identified 1,165 isolates, 73.7% were *M*. *tuberculosis* and 26.2%, *M*. *bovis*. Among pulmonary samples, 16.6% were *M*. *bovis*. The proportion of *M*. *bovis* isolates significantly increased from 7.8% in 2000 to 28.4% in 2014 (*X* ^*2*^_trend_, p<0.001). Primary STR resistance was higher among *M*. *bovis* compared with *M*. *tuberculosis* isolates (10.9% vs.3.4%, p<0.001). Secondary multidrug resistance (MDR) rates were 38.5% and 34.4% for *M*. *bovis* and *M*. *tuberculosis*, respectively (p = 0.637). A rising trend of primary STR monoresistance was observed for both species (3.4% in 2000–2004 vs. 7.6% in 2010–2014; p = 0.02).

**Conclusions/Significance:**

There is a high prevalence and a rising trend of *M*. *bovis* isolates in our region. The proportion of pulmonary *M*. *bovis* isolates is higher than in previous reports. Additionally, we report high rates of primary anti-tuberculosis resistance and secondary MDR in both *M*. *tuberculosis* and *M*. *bovis*. This is one of the largest reports on drug susceptibility of *M*. *bovis* from human samples and shows a significant proportion of first-line anti-tuberculosis drug resistance.

## Author Summary

Human tuberculosis caused by *Mycobacterium bovis* (HTBMb) is a lesser-known form of the disease. The main route of transmission of HTBMb is the consumption of unpasteurized dairy products, causing mostly extrapulmonary disease. *M*. *bovis* is naturally resistant to pyrazinamide, a drug that allows for a shorter treatment course. Therefore, if *M*. *bovis* is not properly identified or if there is resistance to other drugs, proper treatment may be hindered. Most laboratories in developing countries do not routinely perform mycobacterial cultures, and only a few laboratories can identify *M*. *bovis*. Therefore, HTBMb cases are believed to be underestimated. We report a large proportion of *M*. *bovis* isolates and an increasing isolation trend across time. We report a large proportion of *M*. *bovis* isolates from pulmonary samples, suggesting the possibility of human-to-human airborne transmission. Also, we showed that *M*. *bovis* isolates were more frequently resistant to streptomycin, perhaps as a result of antibiotic usage in cattle. This work underscores the need for identification to the species level, proper susceptibility testing, as well as a stricter control of bovine tuberculosis.

## Introduction

Tuberculosis (TB) remains an important health problem in several regions of the world, especially in countries with a high prevalence of HIV infection. *Mycobacterium tuberculosis* complex (MTBC) includes closely related species among which *M*. *tuberculosis*, *M*. *bovis*, and *M*. *africanum* are the most frequently associated with human disease. [1] Unlike *M*. *tuberculosis*, *M*. *bovis* can infect a broad range of mammals, including cattle and, therefore, it is considered a zoonosis. The main mechanism of contagion in humans is the consumption of unpasteurized dairy products and, less frequently, animal-to-human and human-to-human contact. [2–4]

Historically, the burden of human TB caused by *M*. *bovis* (HTBMb) has been closely related to that of bovine TB (BTB) in the same region.[5] Unfortunately, data from most developing countries, where there is still inappropriate BTB control, is scarce.[6,7] There are several factors that explain the underreporting of HTBMb in these regions. First, control programs rely on acid-fast bacilli (AFB) smears as the primary diagnostic method in suspected TB cases; and mycobacterial culture is performed only when drug resistance is suspected, or treatment is failing. Second, most laboratories use culture medium containing glycerol, and this reduces the probability of *M*. *bovis* isolation. Lastly, only a few laboratories can identify MTBC at the species level.[8,9] In Mexico, one national reference laboratory (Instituto Nacional de Diagnóstico y Referencia Epidemiológicos) and 32 public health laboratories are capable of performing mycobacterial cultures. However, species-level identification is not routinely performed. Consequently, data regarding first-line anti-tuberculosis drug susceptibility for MBTC are scarce. Therefore, describing the burden of HTBMb and first-line drug susceptibility pattern has important implications for treatment, referral, and public health policies in developing countries. We describe the trends of *M*. *bovis* isolation and first-line drug susceptibility for a referral laboratory in a tertiary care hospital during a 15-year period.

## Methods

This study was conducted at Instituto Nacional de Ciencias Médicas y Nutrición Salvador Zubirán, one of the National Institutes of Health in Mexico. This tertiary care referral center accepts adult patients from all over the country. The patient population involves complex medical and surgical cases including rheumatologic patients, bone marrow and solid organ transplant recipients, and HIV patients. The Laboratory of Clinical Microbiology receives local and referred clinical samples from other National Institutes of Health as well as other hospitals in Mexico City and nearby states for mycobacterial culture. Since 1992, as a regular practice, samples from patients suspected to have TB undergo mycobacterial culture, species-level identification and first-line drug susceptibility testing in all instances. We selected the 2000–2014 period because laboratory practices for mycobacterial isolation, identification, and susceptibility testing became more uniform then.

### Ethics statement

Institutional approval for this study was obtained from the Comité Institucional de Investigación Biomédica en Humanos. All data analyzed were anonymized.

### Data collection and definitions

A search was performed using the Laboratory of Clinical Microbiology database to investigate all MTBC isolates from January 2000 to December 2014. This database includes both local and referred samples. Only the first isolate was considered for the analysis when multiple samples within a 6-month period were available for a single patient. Whenever there were additional isolates from the same patient ≥ 6 months apart, they were considered as separate episodes and included for analysis.

Data on mycobacterial species, drug susceptibility, sample source, treatment, and referral status were obtained. HIV status was obtained for local samples only. Primary resistance (new cases) was defined as those samples from patients without previous anti-tuberculosis treatment. Secondary resistance (treated cases) was defined as those samples from patients who had previously received any anti-tuberculosis treatment. Resistance to both isoniazid (INH) and rifampin (RIF) was defined as multidrug resistance (MDR). Polydrug resistance was defined as resistance to two or more drugs but excluding those classified as MDR. Samples from sputum, bronchoalveolar lavage, endotracheal aspirate, gastric aspirate, lung and pleural biopsy, and pleural fluid were classified as pulmonary. Liver, spleen, and gastrointestinal biopsies, as well as ascites fluid, and fecal samples were classified as abdominal. Additionally, if samples from the same patient were obtained from a pulmonary and an extrapulmonary source, the case was classified as pulmonary.

### Microbiology procedures

As a laboratory standard procedure, all samples obtained from bronchoalveolar lavage, cerebrospinal fluid, and biopsies from any tissue or abscess were cultured in mycobacteria-specific culture medium, regardless of TB suspicion. Sputum and urine samples were cultured for mycobacteria at the request of the treating physician.

Samples were digested and decontaminated by the NALC-NaOH method as previously described. [10,11] After digestion, samples were inoculated in both Löwenstein-Jensen medium and MGIT tubes (Becton-Dickinson, Sparks, MA, USA) according to the manufacturer’s specifications. Biopsy samples were additionally inoculated in Stonebrink culture medium. Additionally, smears from all samples were prepared for Ziehl-Neelsen and Auramine-rhodamine stain. Isolates obtained from MGIT tubes were sub-cultured in Stonebrink and Löwenstein-Jensen medium. All positive cultures were further identified by DNA probe (Accuprobe, GEN-PROBE, San Diego, CA). Biochemical tests (niacin production, nitrate reduction, thiophen-2-carboxylic acid anhydride susceptibility, and pyrazinamidase deamidation) for the identification of *M*. *bovis* were performed in those positive cultures with dysgonic growth. [12] Spoligotyping was performed for local isolates as previously described, and data was entered into an international database (www.mbovis.org).[13] Susceptibility testing for anti-tuberculosis drugs was performed for INH, RIF, streptomycin (STR) and ethambutol (EMB). For this purpose, and according to the manufacturer’s specifications, the radiometric BACTEC 460 TB culture system (Becton-Dickinson, Sparks, MA, USA) was used from the years 2000 to 2010 with the following drug concentrations: INH (0.1 μg/mL), RIF (2.0 μg/mL), EMB (7.5 μg/mL), and STR (6.0 μg/mL); then, from 2010 on, the BACTEC MGIT 960 culture system (Becton-Dickinson, Sparks, MA, USA) was used with the following concentrations: INH (0.1 μg/mL), RIF (1.0 μg/mL), EMB (5.0 μg/mL), STR (2.0 μg/mL). This laboratory is subjected to regular quality control evaluations by the Centers for Disease Control and Prevention, and the College of American Pathologists for identification and susceptibility testing of mycobacteria species.

### Statistical analysis

Statistical analysis was performed using STATA 11.0 software (StataCorp, College Station, TX, USA). Categorical data was summarized using frequency tables, and the X^2^ test was used for comparison between groups. The *M*. *bovis* case proportion by year was analyzed obtaining a X^2^ for trend by the Armitage test (regression). A p-value <0.05 was determined as statistically significant for all tests.

## Results

### Mycobacterial species and patient characteristics

**Fig 1:**
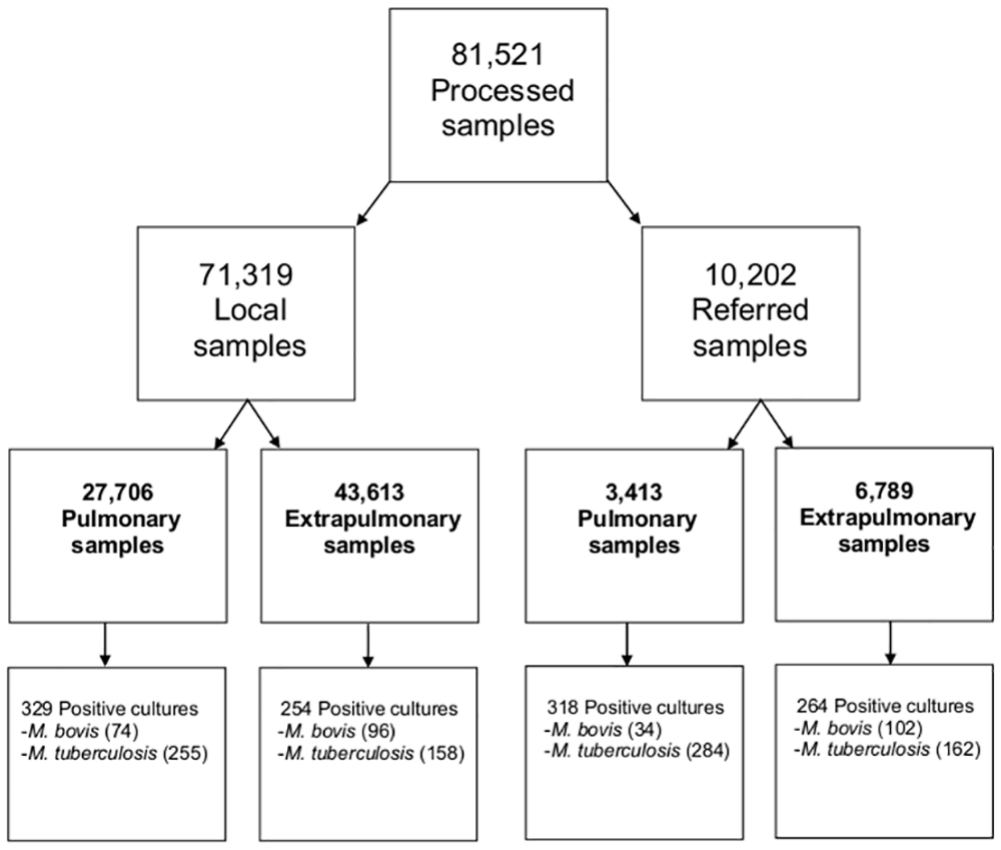
Samples processed for mycobacterial culture during the study period.

During the study period, 81,521 samples were processed for mycobacterial culture (Fig 1). Among these, 1,165 MTBC isolates were identified, 583 (50.0%) as local samples and 582 (49.9%) as referrals after eliminating duplicate cultures. Of these, 73.7% (859/1,165) were identified as *M*. *tuberculosis*, and 26.2% (306/1,165) as *M*. *bovis*. Sixty-two percent of the *M*. *tuberculosis* isolates and 35.2% of the *M*. *bovis* isolates were obtained from pulmonary samples (p<0.001; Table 1). Data on AFB stain were available for 954 isolates; the proportion of positive AFB stain was 75.9% (378/680) for *M*. *tuberculosis*, and 24.1% (120/274) for *M*. *bovis* (p = 0.001). Among the AFB-positive *M*. *bovis* isolates, 45.8% (55/120) were pulmonary, and 54.1% (65/120) were extrapulmonary (p = 0.018). Conversely, among the AFB-positive *M*. *tuberculosis* isolates, 79.3% (300/378) were pulmonary and 20.6% (78/378) were extrapulmonary (p<0.001).

**Table 1:** Anatomical Site of Isolation.

Site	Total (N = 1,165)	*M*. *tuberculosis* (n = 859)	*M*. *bovis* (n = 306)	p*
	N (%)	n (%)	n (%)	
Pulmonary^1^	647(55.5)	539 (62.7)	108 (35.3)	<0.001
Bone, joint, skin, and soft-tissue	51 (4.4)	35 (4.1)	16 (5.2)	0.397
Genitourinary	73 (6.3)	51 (5.9)	22 (7.2)	0.438
Blood or bone marrow	39 (3.3)	34 (4)	5 (1.6)	0.052
Cerebrospinal fluid	95 (8.2)	58 (6.8)	37 (12.1)	0.003
Abdominal^2^	55 (4.7)	26 (3)	29 (9.5)	<0.001
Lymph node	138 (11.8)	78 (9.1)	60 (19.6)	<0.001
Other	67 (5.8)	38 (4.4)	29 (9.5)	0.001
Total extrapulmonary	518 (44.4)	320 (37.2)	198 (64.7)	<0.001

NOTE.

* X^2^ test.

^1^ Sputum, bronchoalveolar lavage, endotracheal aspirate, gastric aspirate, lung and pleural biopsy, and pleural fluid.

^2^ Liver, spleen, and gastrointestinal biopsies, ascites fluid, and fecal samples.

One hundred and twelve (19.2%) local isolates were from HIV-infected patients; 63.3% (71/112) were *M*. *tuberculosis*, and 36.6% (41/112) were *M*. *bovis* (p = 0.054). Among the samples from HIV-infected patients, 52.6% (59/112) were pulmonary samples. Of these, 76.2% (45/59) were identified as *M*. *tuberculosis*, and 23.7% (14/59) were *M*. *bovis*.

**Fig 2:**
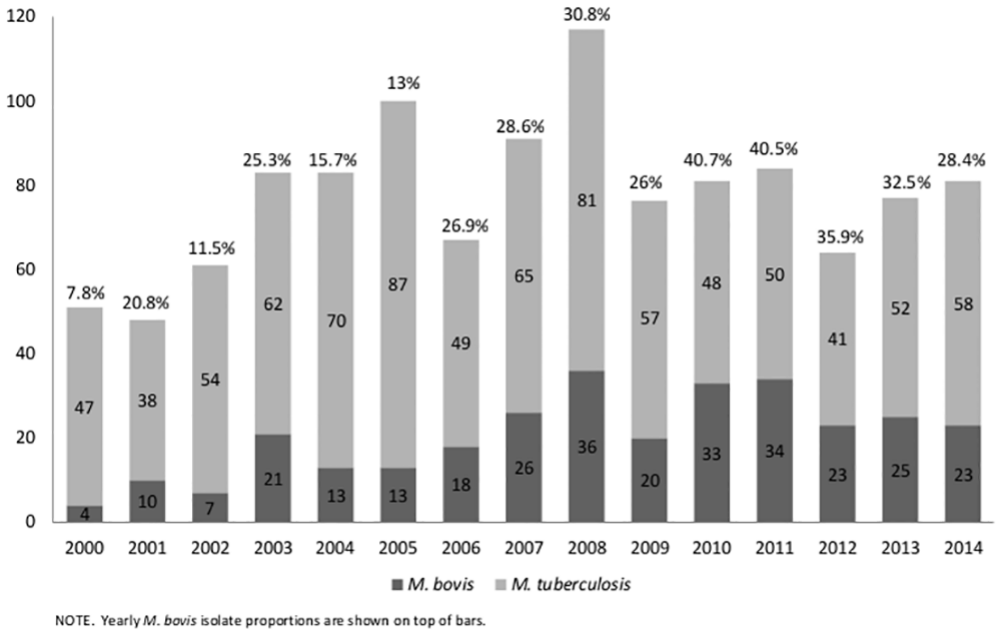
*Mycobacterium tuberculosis* complex Isolates per year and *M*. *bovis* proportions.

The overall proportion of *M*. *bovis* isolation significantly increased from 7.8% in 2000 to 28.4% in 2014 (*X* ^*2*^_trend_, p<0.001; Fig 2). Spoligotype pattern was available for 63.5% (108/170) of the local samples (S1 Table).

### Susceptibility to first-line anti-tuberculosis drugs

Data on first-line anti-tuberculosis drugs susceptibility were available for 1,139 (97.7%) isolates (Table 2). When considering monoresistance among all isolates, 10.9% of *M*. *bovis* and 3.2% of *M*. *tuberculosis* were resistant to STR (p<0.001). This association remained after stratifying by new and treated cases (p<0.001 and p = 0.032, respectively). Total MDR among all cases was 11.9% for *M*. *tuberculosis* and 7.6% for *M*. *bovis* (p = 0.038). This same association was observed among new cases (6.8% vs. 3%, p = 0.026). However, among treated cases no difference was observed.

**Table 2:** First-line drug resistance profile.

		All cases n (%)				Primary resistance (New cases) n (%)				Secondary resistance (Treated cases) n (%)		
Resistance profile	Total N = 1,139	*M*. *tuberculosis* n = 835	*M*. *bovis* n = 304	p*	Total n = 946	*M*. *tuberculosis* n = 681	*M*. *bovis* n = 265	P	Total n = 193	*M*. *tuberculosis* n = 154	*M*. *bovis* n = 39	p
Any drug resistance												
INH	228 (20)	179 (21.4)	49 (16.1)	**0.047**	135 (14.3)	104 (15.3)	31 (11.7)	0.158	93 (48.2)	75 (48.7)	18 (46.2)	0.776
RIF	143 (12.6)	114 (13.7)	29 (9.5)	0.064	72 (7.6)	58 (8.5)	14 (5.3)	0.092	71 (36.8)	56 (36.4)	15 (38.5)	0.808
EMB	27 (2.4)	25 (3)	2 (0.7)	**0.022**	14 (1.5)	13 (1.9)	1 (0.4)	0.080	13 (6.7)	12 (7.8)	1 (2.6)	0.245
STR	120 (10.5)	84 (10.1)	36 (11.8)	0.386	90 (9.5)	59 (8.7)	31 (11.7)	0.153	30 (15.5)	25 (16.2)	5 (12.8)	0.599
Total any drug resistance	309 (27.1)	221 (26.5)	88 (28.9)	0.405	205 (21.7)	139 (20.4)	66 (24.9)	0.132	104 (53.9)	82 (53.2)	22 (56.4)	0.723
Monoresistance												
INH	77 (6.8)	54 (6.5)	23 (7.6)	0.514	57 (6)	36 (5.3)	21 (7.9)	0.126	20 (10.4)	18 (11.7)	2 (5.1)	0.230
RIF	19 (1.7)	13 (1.6)	6 (2)	0.627	16 (1.7)	10 (1.5)	6 (2.3)	0.394	3 (1.6)	3 (1.9)	0 (0)	--
EMB	--	--	--	--	--	--	--	--	--	--	--	--
STR	60 (5.3)	27 (3.2)	33 (10.9)	**<0.001**	52 (5.5)	23 (3.4)	29 (10.9)	**<0.001**	8 (4.1)	4 (2.6)	4 (10.3)	**0.032**
Total monoresistance	156 (13.7)	94 (11.3)	62 (20.4)	**<0.001**	125 (13.2)	69 (10.1)	56 (21.1)	**<0.001**	31 (16.1)	25 (16.2)	6 (15.4)	0.897
Multidrug resistance												
INH+RIF	76 (6.6)	55 (6.5)	21 (6.9)	0.848	34 (3.5)	27 (3.9)	7 (2.6)	0.326	42 (21.7)	28 (18.1)	14 (35.9)	**0.017**
INH+RIF+EMB	15 (1.3)	13 (1.6)	2 (0.7)	0.239	6 (0.6)	5 (0.7)	1 (0.4)	0.535	9 (4.7)	8 (5.2)	1 (2.6)	0.486
INH+RIF+STR	24 (2.1)	24 (2.9)	0 (0)	--	10 (1.1)	10 (1.5)	0 (0)	--	14 (7.3)	14 (9.1)	0 (0)	--
INH+RIF+EMB+STR	7 (0.6)	7 (0.8)	0 (0)	--	4 (0.4)	4 (0.6)	0 (0)	--	3 (1.6)	3 (1.9)	0 (0)	--
Total multidrug resistance	122 (10.7)	99 (11.9)	23 (7.6)	**0.038**	54 (5.7)	46 (6.8)	8 (3)	**0.026**	68 (35.2)	53 (34.4)	15 (38.5)	0.637
Polydrug resistance												
INH+EMB	2 (0.2)	2 (0.2)	0 (0)	--	2 (0.2)	2 (0.3)	0 (0)	--	0 (0)	0 (0)	0 (0)	--
INH+STR	25 (2.2)	22 (2.6)	3 (1)	0.093	21 (2.2)	19 (2.8)	2 (0.8)	0.056	4 (2.1)	3 (1.9)	1 (2.6)	0.809
RIF+STR	1 (0.1)	1 (0.1)	0 (0)	--	1 (0.1)	1 (0.1)	0 (0)	--	0 (0)	0 (0)	0 (0)	--
RIF+EMB+STR	1 (0.1)	1 (0.1)	0 (0)	--	1 (0.1)	1 (0.1)	0 (0)	--	0 (0)	0 (0)	0 (0)	--
Total polydrug resistance	31 (2.7)	28 (3.4)	3 (1)	**0.03**	26 (2.7)	24 (3.5)	2 (0.8)	**0.019**	5 (2.6)	4 (2.6)	1 (2.6)	0.991

NOTE.

* X^2^ test. INH.—Isoniazid; RIF.-. Rifampin; EMB.—Ethambutol; STR.–Streptomycin.

An increasing trend of STR monoresistance among new cases was found when considering both species (3.4% in 2000–2004 vs. 7.6% in 2010–2014, p = 0.02) (Table 3).

**Table 3:** Primary resistance trends of *Mycobacterium tuberculosis* complex across time.

		Total %				*M*. *tuberculosis* %				*M*. *bovis* %		
Year	2000–2004	2005–2009	2010–2014	p*	2000–2004	2005–2009	2010–2014	p	2000–2004	2005–2009	2010–2014	p
No. isolates tested	258	335	353		213	238	230		45	97	123	
Drug resistance pattern												
Any drug resistance												
Any resistance to INH	10.1	16.2	10.4	0.874	10.9	17.1	11.2	0.963	6.3	14.2	8.9	0.977
Any resistance to RIF	4.1	10.7	5.6	0.690	4.5	12.2	6.1	0.573	2.2	6.7	4.7	0.755
Any resistance to EMB	0.8	2.0	1.4	0.595	0.9	2.9	1.7	0.567	0	0	0.8	0.341
Any resistance to STR	6.2	8.0	11.1	**0.023**	6.2	7.4	10.2	0.103	6.3	9.3	12.8	0.174
Total any drug resistance	13.4	20.2	18.5	0.119	13.4	19.9	17.0	0.308	13.5	21.1	21.2	0.320
Monoresistance												
Monoresistance to INH	4.8	6.4	5.6	0.713	4.5	5.6	5.0	0.824	6.3	8.5	6.8	0.959
Monoresistance to RIF	0.8	1.8	2.2	0.172	0.5	2.1	1.7	0.283	2.2	1.0	3.1	0.501
Monoresistance to STR	3.4	4.0	7.6	**0.012**	2.7	2.1	5.0	0.169	6.3	8.5	12.1	0.191
Total monoresistance	8.5	11.4	14.1	**0.023**	7.4	9.2	10.9	0.187	13.5	16.4	19.6	0.281
Multidrug resistance												
INH+RIF	2.3	6.9	1.4	0.333	2.8	7.1	1.7	0.514	0	6.2	0.8	0.583
INH+RIF+EMB	0.4	0.9	0.6	0.839	0.5	1.2	0.4	0.945	0	0	0.8	0.341
INH+RIF+STR	0.8	1.5	0.8	0.999	0.9	2.1	1.3	0.770	0	0	0	--
INH+RIF+EMB+STR	0	0.9	0.3	0.708	0	1.2	0.4	0.577	0	0	0	--
Total Multidrug resistance	3.4	9.2	3.0	0.566	4.1	10.5	3.8	0.837	0	5.8	1.6	0.866

NOTE.

* X^2^ test for trend. INH.—Isoniazid; RIF.-. Rifampin; EMB.—Ethambutol; STR.–Streptomycin.

## Discussion

This report demonstrates a high prevalence and a rising trend in the proportion of *M*. *bovis* isolation in our laboratory. This report is one of the largest on first-line anti-tuberculosis drug profile of *M*. *bovis* and shows a noteworthy proportion of first-line anti-tuberculosis drug resistance and secondary MDR isolates.

The proportion of *M*. *bovis* isolates in this study (26.2%) is much higher than that reported by other hospital-based studies in Latin America (0.4%) and by other hospitals in Mexico (<1%). [14,15] This may be explained by the larger study period of the present report and the high proportion of samples from immunosuppressed patients who are at a greater risk for *M*. *bovis* infection, as documented in previous studies.[16] In fact, isolates obtained from HIV-infected patients accounted for 19.2% of the local samples.[17]

We also identified a rising trend in the proportion of cases caused by *M*. *bovis* across time. HMBTb is considered a reflection of the BTB burden in the region. In fact, we recently identified a high burden of bovine and human TB in a dairy production facility in rural Mexico. [2] This also correlates with previous reports of *M*. *bovis* among artisanal dairy products, which have been linked to HMBTb cases in Mexico and along the south border cities of the United States.[18,19] Mexico is considered a country of “sporadic occurrence” of BTB by the World Organization for Animal Health. However, like in many countries in the region, the test-and-slaughter strategy for bovine tuberculosis control is not universally implemented. [6] The main obstacles for BTB control in Mexico are financial and cultural. Official government data reports an overall prevalence of BTB of 2.05% in 2015, but it reaches 16.5% among dairy farms.[20] The reason for this difference may be explained by the fact that meat producing regions require to be certified as BTB free for cattle export. On the contrary, in dairy production farms, BTB only mildly affects production and pasteurization eliminates *M*. *bovis*. [21] Unfortunately, about 30% of the milk production in Mexico is sold without pasteurization, mostly to small retailers and artisan cheese producers.[22]

The respiratory route of contagion is considered less efficient for *M*. *bovis* than for *M*. *tuberculosis*. However, recent data detailing outbreaks in the community and hospitals demonstrated that human-to-human contagion is not as unlikely as previously believed.[3,23,24] Interestingly, we observed an important proportion (16.6%) of *M*. *bovis* isolates recovered from pulmonary samples from a mainly urban population. Therefore, it may be hypothesized that airborne human-to-human transmission of *M*. *bovis* may occur in the community, but remains undetected in our region given that mycobacterial culture is not routinely performed. Unfortunately, as an important limitation of this study, the lack of clinical and epidemiologic data precludes us from reaching a definite conclusion.

We also report a high rate of first-line anti-tuberculosis resistance for MBTC. The proportion of INH (14%), RIF (7.6%), and STR (5.5%) primary resistance found among new cases is considerably higher for INH and RIF than in previous reports from our group in 1995 (INH 6%, RIF 2%, STR 6%).[25] These proportions are also higher than those from other reports in Mexico (1995 to 2006) among new cases (INH 9%, RIF 3%, and MDR 4.5%), and are also higher than recent data from the National Survey on TB Drug Resistance in Mexico (INH 3.5%, RIF 0.1%, STR 4% and MDR 2.3%).[26,27] This discrepancy may be explained by the different periods from which data was collected and the dissimilar patient population (ours being hospital-based and including more HIV-infected, and immunosuppressed patients).

When comparing the resistance profiles of *M*. *bovis* and *M*. *tuberculosis*, we observed a considerably higher primary MDR *M*. *tuberculosis* proportion, and a significantly higher STR monoresistance among *M*. *bovis* isolates. Data regarding drug susceptibility for *M*. *bovis* TB in humans and animals is limited. A study from San Diego reported 7% resistance for INH and 1% for RIF among 167 *M*. *bovis* TB cases.[28] Drug susceptibility has also been reported from outbreaks caused by MDR strains; however, most other case series of HTBMb report full susceptibility to all first-line anti-tuberculosis drugs.[14,15,29–32] A report from the National TB Genotyping Service of the United States informed of 17% of STR resistance among 165 *M*. *bovis* isolates; however, no explanation for this was proposed. [33] Drug susceptibility from *M*. *bovis* isolates collected from farm animals or wildlife is almost uniformly reported as fully susceptible to anti-tuberculosis drugs.[34–36]. We believe that a high proportion of primary resistance for STR among *M*. *bovis* isolates, may be explained by the use of aminoglycosides for treating other diseases in cattle.[37] Some have suggested that primary resistance to INH and RIF in *M*. *bovis* may indicate human-to-human transmission.[32] However, we surveyed BTB in a dairy farm, where samples from cows were obtained during necropsy. We recovered 150 *M*. *bovis* isolates; among these, we observed an even higher rate of STR resistance (15.6%) and a similar rate of INH (9.2%) and RIF (3.4%) resistance. (M. Bobadilla, Instituto Nacional de Ciencias Médicas y Nutrición Salvador Zubirán, Mexico City, personal communication).

It has been suggested that HTBMb cases may be at a higher risk for developing MDR strains if natural resistance to pyrazinamide is not considered and monoresistance to INH or RIF is present. [38,39] We identified a similar proportion of secondary MDR *M*. *bovis* and *M*. *tuberculosis* isolates. Unfortunately, only a few cases were analyzed and no data on previous treatments or outcomes were available, therefore we are unable to conclude if this assumption is true. Nevertheless, it should be recognized that TB cases caused by MDR *M*. *bovis* may result in disease that is harder to treat on a second- line drug regimen. This highlights the need for performing species-level identification and drug susceptibility testing whenever *M*. *bovis* is suspected.

In conclusion, we believe that data contained in this study is relevant in terms of public health and highlights the need for more stringent control of BTB in our country. It also underscores the importance of proper identification of *M*. *bovis* given that the considerable rate of primary resistance to INH and RIF along with the natural pyrazinamide resistance may result in treatment failures and select for MDR strains.

## Supporting Information

S1 Table Spoligotypes of *Mycobacterium bovis* isolates from Human Samples in a Tertiary Care Hospital in Mexico City: 2000–2014. (DOCX)

S1 Checklist STROBE Checklist. (DOC)
