# Supplementary material for: Trends of Mycobacterium bovis Isolation and First-Line Anti-tuberculosis Drug Susceptibility Profile: A Fifteen-Year Laboratory-Based Surveillance
Source: PLoS Negl Trop Dis. 2015 Sep 30;9(9):e0004124. doi: 10.1371/journal.pntd.0004124 (PMC4589280; doi:10.1371/journal.pntd.0004124)
Supplement: S1 Table — (DOCX) [file pntd.0004124.s001.docx]

| SB number | n/N (%) | Octal number |
| --- | --- | --- |
| SB0120 | 1/108 (0.9) | 676773777777600 |
| SB0121 | 7/108 (6.4) | 676773677777600 |
| SB0130 | 2/108 (1.8) | 676573777777600 |
| SB0140 | 16/108 (14.8) | 664073777777600 |
| SB0145 | 11/108 (10.1) | 640013777777600 |
| SB0269 | 1/108 (0.9) | 664063777777600 |
| SB0327 | 4/108 (3.7) | 676573777077600 |
| SB0669 | 4/108 (3.7) | 264063777777600 |
| SB0673 | 26/108 (24.1) | 264073777777600 |
| SB0971 | 11/108 (10.2) | 664073777700600 |
| SB0986 | 1/108 (0.9 | 264071777777600 |
| SB1040 | 1/108 (0.9 | 640013377777600 |
| SB1165 | 2/108 (1.8) | 640033777777600 |
| SB1211 | 1/108 (0.9) | 640013017777600 |
| SB1495 | 2/108 (1.8) | 640012017777600 |
| SB1754 | 1/108 (0.9) | 664073400007600 |
| SB1936 | 1/108 (0.9) | 676573777043600 |
| SB2084 | 1/108 (0.9) | 656463737777600 |
| SB2117 | 1/108 (0.9) | 064043777777600 |
| Not assigned | 1/108 (0.9) | 074573777777600 |
| Not assigned | 1/108 (0.9) | 240033777777600 |
| Not assigned | 1/108 (0.9) | 264073777077600 |
| Not assigned | 4/108 (3.7) | 264073777760600 |
| Not assigned | 1/108 (0.9) | 440001777777600 |
| Not assigned | 1/108 (0.9) | 464077377777600 |
| Not assigned | 1/108 (0.9) | 650673377777600 |
| Not assigned | 1/108 (0.9) | 664073400007600 |
| Not assigned | 1/108 (0.9) | 664073757607600 |
| Not assigned | 1/108 (0.9) | 664073777760600 |
| Not assigned | 1/108 (0.9) | 676773634777600 |

Note: Data obtained from www.mbovis.org
